# Development of a Mobile Clinical Prediction Tool to Estimate Future Depression Severity and Guide Treatment in Primary Care: User-Centered Design

**DOI:** 10.2196/mhealth.9502

**Published:** 2018-04-23

**Authors:** Caroline Wachtler, Amy Coe, Sandra Davidson, Susan Fletcher, Antonette Mendoza, Leon Sterling, Jane Gunn

**Affiliations:** ^1^ Department of Neurobiology Karolinska Institutet Solna Sweden; ^2^ Department of General Practice The University of Melbourne Carlton Australia; ^3^ Computing and Information Systems The University of Melbourne Parkville Australia; ^4^ Centre for Design Innovation Swinburne University of Technology Hawthorn Australia

**Keywords:** user-centered design, depression, ehealth, primary health care, decision support techniques, risk, mental health

## Abstract

**Background:**

Around the world, depression is both under- and overtreated. The *diamond* clinical prediction tool was developed to assist with appropriate treatment allocation by estimating the 3-month prognosis among people with current depressive symptoms. Delivering clinical prediction tools in a way that will enhance their uptake in routine clinical practice remains challenging; however, mobile apps show promise in this respect. To increase the likelihood that an app-delivered clinical prediction tool can be successfully incorporated into clinical practice, it is important to involve end users in the app design process.

**Objective:**

The aim of the study was to maximize patient engagement in an app designed to improve treatment allocation for depression.

**Methods:**

An iterative, user-centered design process was employed. Qualitative data were collected via 2 focus groups with a community sample (n=17) and 7 semistructured interviews with people with depressive symptoms. The results of the focus groups and interviews were used by the computer engineering team to modify subsequent protoypes of the app.

**Results:**

Iterative development resulted in 3 prototypes and a final app. The areas requiring the most substantial changes following end-user input were related to the iconography used and the way that feedback was provided. In particular, communicating risk of future depressive symptoms proved difficult; these messages were consistently misinterpreted and negatively viewed and were ultimately removed. All participants felt positively about seeing their results summarized after completion of the clinical prediction tool, but there was a need for a personalized treatment recommendation made in conjunction with a consultation with a health professional.

**Conclusions:**

User-centered design led to valuable improvements in the content and design of an app designed to improve allocation of and engagement in depression treatment. Iterative design allowed us to develop a tool that allows users to feel hope, engage in self-reflection, and motivate them to treatment. The tool is currently being evaluated in a randomized controlled trial.

## Introduction

### Background

Depression affects at least 350 million people worldwide [1]. Primary care doctors are responsible for most of the identification, treatment, and management of depression [2], with between 24% and 55% of primary care attendees reporting depressive symptoms [3]. Research shows that with appropriate treatment, recovery from depression is possible [4]. However, there is frequently a mismatch between patient needs and the treatment they receive. Patients with subthreshold or mild depression who are likely to recover spontaneously are often overtreated [5], whereas patients with severe symptoms frequently do not receive minimally adequate treatment [4,6,7]. Treatment mismatch is associated with poor patient outcomes and represents an inefficient distribution of scarce resources [8,9]. Currently, there is no systematic way of matching patients with depressive symptoms with the most appropriate level of treatment in primary care.

To improve treatment allocation for depression, we developed the *diamond* clinical prediction tool that is designed to assess an individual’s future depressive symptom severity and provide them with an evidence-based treatment recommendation matched to their prognosis. Details of the clinical prediction tool’s development are available elsewhere [10]; briefly, it was developed after an extensive search of the literature identified no existing tools that predicted future symptom severity and could be delivered at scale in the primary care setting. Despite their potential to improve health care [11], relatively few clinical prediction tools have been successfully incorporated into routine clinical practice [12]. Doctors report that time constraints and difficulty of use and interpretation are key barriers to the implementation of clinical prediction tools [13]. Delivering a clinical prediction tool directly to patients via a digital app has the potential to overcome these barriers and increase the use of clinical prediction tools in clinical practice. Moreover, studies suggest that patient-completed tools can increase patient participation in their own health care and increase the efficiency of health care encounters [14-16].

### eHealth and User-Centered Design

Despite the wide availability of apps, they have yet to revolutionize health care, due in part to lack of uptake. User attrition from or nonadherence to electronic health (eHealth) technologies is well documented, both for patients and clinicians [17,18]. The few existing implementation studies of specific eHealth decision support technologies have identified several barriers, including low user acceptance, poor face validity, and low user-friendliness [19,20]. To successfully change health care practices, eHealth technologies must be engaging to end users, deliver easily understood information, and promote engagement with any treatment recommendation they may provide.

Explicit user-centered design, a process in which end users influence how a design takes shape [21], may improve the chances of successful implementation of technology in practice. Apps developed using this process have reported improved user acceptance, face validity, user-friendliness, and uptake [22-25]. User-centered design is based on the principles of participatory design and involves all potential stakeholders. It uses iterative formative evaluation during the entire development process and accounts for the conditions of implementation from the beginning [15,23]. In health care, the end user of a technology may be the patient, but ideally, user-based technology development should identify and take into account all potential stakeholders including clinicians, researchers responsible for the content of the technology, and representatives of the health care system [16].

### This Study

In this study, we describe the user-centered design process of an app to assess individual risk of persistent depressive symptoms and recommend individually tailored treatment based on current knowledge about best-evidence treatment for depression. Our aim was to focus on users’ emotional and cognitive experience to design an acceptable tool for clinical decision support. Users were involved to determine

1. How the tool should look (to ensure it was credible, easy to use, and visually attractive)
2. What feedback was most likely to promote engagement with treatment recommendations
3. How the feedback should be presented.

## Methods

### User-Centered Design

User-centered design is an umbrella term that encompasses a range of models and approaches that software developers can employ to produce a highly usable and accessible product [26]. The term was coined in the late 1990s by Donald Norman who posited that three levels of cognitive processing should be considered in designing useable products: (1) *visceral processing*, which refers to the look and feel of a product; (2) *behavioral processing*, which relates to the experience of product characteristics such as performance and usability; and (3) *reflective processing*, which refers to characteristics such as the meaning of a product, its impact on self-image, and satisfaction [27]. Reflective processing has been shown to be most important for adoption and use of a product [21]; the successful implementation of software depends on its ability to address peoples’ values, satisfy their emotional needs and expectations, and to encourage participation, acceptance, and trust [22-24]. For eHealth technologies that have the specific aim of changing clinician and patient attitudes and behaviors [21,28], reflective processing is particularly critical. Therefore, we approached our user-centered design process with Norman’s framework and the importance of reflective processing in mind.

#### Stage 1: Identify End Users and Context

To increase the likelihood that the *diamond* clinical prediction tool would be implemented into routine clinical practice, we identified end users and reviewed the environmental characteristics of the context in which it would be used. Environmental characteristics were collated through telephone interviews with primary care attendees experiencing depressive symptoms and discussions with clinicians and primary care researchers and through a literature review of clinical prediction tools in practice. A narrative description of end users and their context was developed.

#### Stage 2: Concept Development

Emotion-driven goal modeling was used to identify requirements based on patient, clinician, and research team goals regarding the app. Emotion-driven goal modeling is based on the theory of agent-oriented modeling [29] and is a method used to identify and interrelate the personal values, motivations, and emotions stakeholders have around software to specify requirements for development. It uses a modified grounded theory approach for analysis of individual interviews, group discussions, and other datasets. For this study, the data for the modeling came from two initial development meetings and five interviews with individuals who had either prior or current experience of depressive symptoms. We also identified the research team’s requirements for the evidence-based content and recommendations. A literature scoping review was conducted to identify the evidence on how to best communicate risk for persistent depression.

#### Stage 3: App Development

Two focus groups, each lasting approximately 2 hours, were conducted by CW and AC. The 10 participants in focus group 1 (1) were presented with icons from the app without any associated text and were requested to write down and verbally present the thoughts and feelings they associated with the image; (2) formed groups of two and took turns using the app prototype on an iPad provided by the research team, followed by general discussion based on semistructured questions; and (3) were presented with the options for risk communication, followed by semistructured interview questions (Textboxes 1 and 2). On the basis of the results from the first focus group, modifications were made to the prototype. Prototype 2 was presented to participants in the second focus group, and the procedure used in the first focus group was repeated. Further modifications to the app were made based on results of focus group 2.

**Topic guide for focus groups 1 and 2.**

1. First impressions
  - What are your first or general impressions of the app?
  - What were the best things about the app?
  - What was the biggest problem you had with the app?
2. Results or feedback
  - How did you feel about the results page?
  - Imagine you’re in your general practitioner (GP) clinic and you complete the app, how would you feel?
3. Risk communication
  - What are your first impressions of (the risk communication)?
  - How do you feel about the faces?
  - How do you feel about the stick figures and numbers?
  - How would you feel if both the stick figures and the faces were presented?
4. Iconography
  - In front of you is a workbook with a picture on each page. For each picture write down what you think each picture represents. We will then discuss as a group.

**Topic guide for individual interviews.**

1. First impressions
  - What were your first impressions of the app?
  - What did you like or dislike?
  - How did the app make you feel?
  - Did the app make you think of any questions or other thoughts when completing it?
2. Results or feedback
  - How did you feel about the message at the end (treatment recommendation)?
  - Was the information clear?

The third prototype of the app was tested in individual interviews. Seven face-to-face semistructured interviews were conducted by CW and observed by AC. Participants were given prototype 3 on an iPad while the observer took notes. After completing the app, they were interviewed using a broad topic guide (Textboxes 1 and 2) covering first impressions of the app and reactions to the feedback and treatment recommendations.

### Data Analysis

The focus groups were audiorecorded. All audio recordings and text produced by participants, moderator, and observers were included in the analysis. AC collated the written and spoken words associated with part 1 of the focus group. CW transcribed the audio recordings. We conducted thematic analysis of the data by iteratively coding individual words, concepts, and phrases and then organizing these codes into a structure of themes and subthemes using the constant comparison method [30]. CW and AC conducted independent coding and then discussed themes and relationships between themes. Because the purpose of this analysis was iterative development of the app, when possible, themes were grouped into features that were requirements of the app. These discussions were relayed back to the research team for discussion of relevance and planning further action.

### Participants

All community-dwelling adults in Melbourne, Australia, who were in the age range of 18 to 65 years and able to respond to recruitment materials in English were eligible for the study. The only exclusion criteria were being outside this age range, and, for participation in an individual interview, the absence of any depressive symptoms (as assessed by the Patient Health Questionnaire-9, PHQ-9 [31]). Participants were recruited through flyers on community noticeboards at The University of Melbourne (Parkville campus) and via advertisements in a weekly university staff e-newsletter, Facebook, and on an online noticeboard website (Gumtree). Participants were sought from the general community, as 87% of the population visits a general practitioner (GP) at least once a year [32]. Focus group recruitment occurred in September (focus group 1) and October (focus group 2) 2014 and individual interview recruitment in February 2015. Interested individuals in the age range of 18 to 65 years were instructed to contact the study coordinator (AC) via phone or email. Upon expression of interest, demographic information was collected for all potential participants. Individuals expressing interest in an individual interview were additionally asked to complete the PHQ-9 at this point, and those with a score of <2 were considered ineligible and not included in the study sample. Participants were given a written plain language statement before participation, and consent was obtained at the time of focus group or interview attendance. Participants received an $50 AUD gift voucher for their time.

### Ethical Approval

This study was approved by the University of Melbourne Human Research Ethics Committee (1442318, 1442584).

## Results

### Participant Characteristics

In total, 17 individuals participated in two focus groups (10 in focus group 1 and 7 in focus group 2), and 7 participated in individual interviews. The demographic characteristics of each group of participants are presented in Table 1. A total of 13 participants were members of the general community, whereas 4 were university staff recruited through campus noticeboards and the staff e-newsletter.

**Table 1:** Characteristics of focus group and interview participants.

Characteristics		Focus group 1 (n=10)	Focus group 2 (n=7)	Interviews (n=7)
**Gender, n**				
	Male	6	4	3
	Female	4	3	4
**Age in years**				
	Range	26-60	25-57	25-45
	Mean (SD)	39.33 (13.36)	39.14 (13.54)	33.14 (7.64)
**Ethnic background, n**				
	White	5	6	6
	Asian	3	0	0
	Hispanic	0	1	0
	Other	2	0	1
**Education, n**				
	Technical and further education	10	2	3
	Bachelor	40	3	2
	Postgraduate	50	2	2

### Stage 1: Identify End Users and Context

End users of the tool were identified as primary care patients and primary care doctors. All primary care patients could use the tool; however, only those whose initial responses to two questions on depressive symptoms indicated that they had depressive symptoms would be taken through to the full assessment and treatment recommendation phases. Our review of the context in which the tool would be used indicated that it should be used by the patient in the waiting room before a consultation with a GP or during the consultation itself. It was believed that this approach was most likely to promote use of the tool, motivate patients to engage in decisions around their health care, and increase the efficiency of the mental health care consultation.

### Stage 2: Concept Development

Emotion goal modeling identified that patients wanted the app to make them feel emotionally supported, and they wanted to feel confident that the information presented to them was relevant and important. Most importantly, users wanted to see the results of their assessment (ie, the risk of having depressive symptoms in 3 months’ time) in a way that was meaningful to them.

Our review of the clinical prediction tool literature identified that a risk communication component, using numerical, verbal, or graphical depictions of risk, is built into most clinical prediction tools. We also identified several challenges in communicating risk to patients: low numerical literacy even in educated populations and the attendant problem of interpretation, considerable margin of error in risk probability, the fact that risk identified by a clinical prediction tool represents a population probability rather than an individualized risk, and ethical issues surrounding the use of risk communication tools as a persuasive mechanism. Adding to these challenges was that, unlike some health problems, the risk probability around persistent depression is very wide, thus increasing the margin of error and the validity of the result. No one type of risk communication emerged as superior to another in communicating risk for persistent depression.

Clinicians wanted to have confidence that the app provided scientifically accurate information, that it looked professional, and that it was useful for improving depression care. Therefore, it was essential that the tool retain, without alteration, all the data items that make up the prognostic algorithm in the *diamond* clinical prediction tool. These questions provided the information necessary to apply the statistical algorithm to predict individual risk for persistent depressive symptoms at 3 months. The required 17 questions for the *diamond* clinical prediction tool include depressive symptom severity as measured by the Patient Health Questionnaire-9 (PHQ-9) [31]; sex; current anxiety; history of depression; presence of chronic illness affecting daily functioning; self-rated health; living alone; and perceived ability to manage on available income (see Table 2). Table 2.

**Table 2:** Items forming the *diamond* clinical prediction tool.

Item number	Text
1	Do you identify more strongly as male or female?
2	In general, would you say your health is
3	Do you have any long-term illnesses, health problem, which limits your daily activities or the work you can do (including problems that are due to old age)?
4	Do you live alone?
5	How do you manage on your available income?
	Over the last 2 weeks, how often have you been bothered by...
6	...Little interest or pleasure in doing things?
7	...Feeling down, depressed or hopeless?
8	...Trouble falling or staying asleep, or sleeping too much?
9	...Feeling tired or having little energy?
10	...Poor appetite or overeating?
11	...Feeling bad about yourself, or that you are a failure, or have let yourself or your family down?
12	...Trouble concentrating on things such as reading the newspaper or watching television?
13	...Moving or speaking so slowly that other people could have noticed. Or the opposite—being so fidgety or restless that you have been moving around a lot more than usual?
14	...Thoughts that you would be better off dead, or of hurting yourself in some way?
15	Have you ever been bothered by feeling down, depressed, or hopeless for longer than 2 weeks?
16	Have you ever been bothered by little interest or pleasure in doing things for longer than 2 weeks?
17	Over the last 4 weeks, how often have you been bothered by feeling nervous, anxious, on edge or worrying a lot about different things?

### Stage 3: App Development

#### Initial Prototype

**Figure 1:**
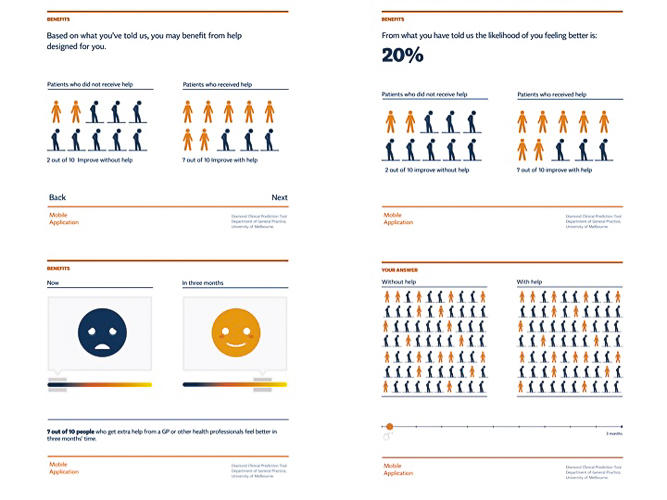
Risk communication presented to participants in prototype 1.

The initial prototype of the tool consisted of three content areas:

1. *Clinical prediction tool items*: see Table 2 for items. Each item was presented on a separate screen, and visual icons intended to represent the item were presented next to the text. Research shows that a mixed format of text and icons enhances comprehension [32] and accuracy of participant responses to questions [33].
2. *A summary of users’ responses*: answers to the clinical prediction tool were reflected back with the text “Things seem to be difficult for you in these areas right now” using the icons described above.
3. *Risk communication*: the patient’s estimated risk of having either mild, moderate, or severe depression in 3 months’ time was presented. To enhance comprehension, risk communication was presented as a comparison between likely mental health outcome in 3 months’ time if the patient did or did not receive treatment. Two alternative ways of presenting risk were presented in the first focus group (see Figure 1).

#### First Impressions

First impressions of the app were positive, with participants reporting it was easy to use, as illustrated in the following quote:

I think the app in general looks quite clean and clinical, for some people a good thing, if they feel they have a problem they want to be handled in a professional way.

Male, 28

The purpose of the app was seen to be to raise awareness of existing mental health problems for the individual, to give hope for improvement, and to motivate the individual to pursue the next step in getting help, as illustrated in the following quotes:

It’s about education, awareness, by answering these questions your becoming aware of some problems you may have, presenting hope, there are things people can do, that you can get better.

Male, 30

If you are this person, then you get this educational fact—these are the areas [you need help with], [this is] how it is impacting you, [it’s] a nudge to get to the next step.

Female, 26

Several participants noted that the “next” button, presented under each question, should be removed to streamline the app, which was agreed upon by the rest of the group.

#### Iconography

There was a clear mismatch for participants between nine of the 12 the visual icons and their intended meaning. For example, participants interpreted an icon depicting a pair of hands as a representation of “charity” or “religion,” when the intended concept was “health.”

#### Summary of User’s Responses

Participants liked having a summary of their results reflected to them. However, they felt that only reflecting the “difficult” areas might have negative consequences, as illustrated in the following quotes:

That’s validation, yeah I feel that way.

Female, 52

You see your problem areas, when you are depressed you just feel bad but this makes it clear.

Female, 30

It could be a negative thing, like look at everything that’s wrong with me...If it were only one or two, that could be identification, but getting a lot back, that could be quite detrimental to some people...It would help to see what was working well.

Male, 26

Participants wanted more meaningful feedback about their results. They wanted an explanation of the severity of the problem, advice on prioritizing areas for attention, and a personalized treatment recommendation based on their results, as illustrated in the following quotes:

Maybe if there is the option to emphasize some problems here it would be better, for example, I’d like to be able to emphasize my sleep problems.

Male, 33

I think there needs to be an answer, to show you have a problem, to contact this GP or call this number, someone to discuss the results.

Male, 26

#### Risk Communication

The risk communication component was identified as the most problematic aspect of the tool. Participants were concerned that presenting risk might make already depressed people feel worse, as illustrated in the following quotes:

If I get help I still have a one in three chance of still feeling bad?

Male, 60

If it has gone 2 months and you are still sick, it’s like there’s only one month left

Male, 26

Participants were confused that the app reported risk at a population level rather than their own personal risk of suffering depression in the future, as illustrated in the following quote:

Impersonal, I’m going to be pigeonholed.

Male, 60

Some participants misinterpreted the message that “with help, you will feel better in three months’ time,” as illustrated in the following quote:

It could be shorter intervals...I mean, if you’re suicidal and you have to wait three months.

Male, 60

All participants misunderstood the risk communication in the form of stick figures, and they felt that it had a negative message, as illustrated in the following quote:

If you are depressed maybe you identify with the sad figure, feels hopeless, not sure this works

Female, 52

Most participants expressed their dislike of the portrayal of risk using emotional faces, as illustrated in the following quotes:

Pretty scary, this looks like a Halloween pumpkin! It’s a bit impersonal.

Male, 60

I feel condescended to [by the animated face].

Female, 53

### Prototype 2

**Figure 2:**
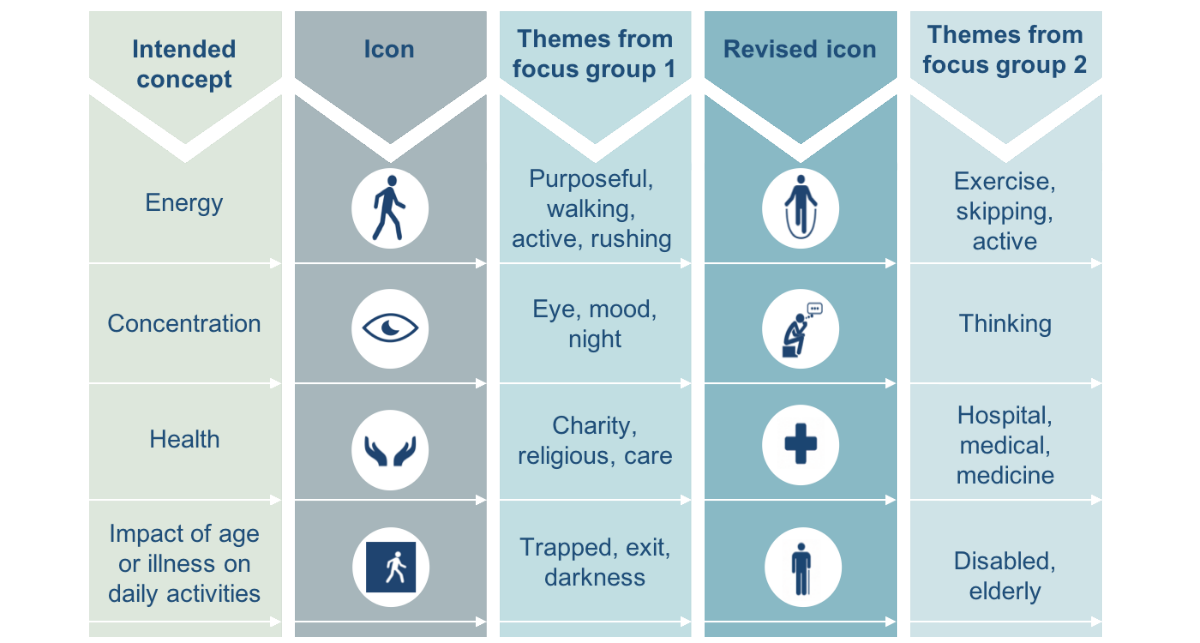
Example of icons replaced and more easily interpreted in prototype 2.

On the basis of the results of focus group 1, a second prototype was developed. The nine most problematic icons were removed and replaced with new icons (see Figure 2 for examples).

The “next” button was removed allowing screens to automatically transition from question to question once a response had been entered. The summary screen was redesigned to reflect areas that “seem to be ok for you right now” in addition to the “difficult” areas presented in prototype 1. Although risk communication was identified as problematic in the first focus group, it was included in the second focus group so that user preferences regarding this component could be explored further. An additional component, treatment recommendation, was added to the end of the tool. This screen informed participants that they could access an online portal with information about mental health treatment options and that a named health care professional was available to talk them through this portal.

#### First Impressions

Similar to focus group 1, participants felt that the second prototype was simple and easy to use. It was seen to be a tool that could guide a conversation with a health provider. Additionally, the app gave an opportunity for the individual to reflect on his or her symptoms, give hope for improvement, and motivate help-seeking. Two participants stated the following:

It would be useful in telling you things you didn’t know, you didn’t think about, and then you’d go into the GP and say, this is right, I’m not sleeping well, it would be a prompt.

Female, 54

This would be a starting point for talking to the GP, for sure, you could focus on what the problems actually are.

Female, 34

#### Iconography

In the second prototype, participants reported that nine of the 12 icons were congruent with the intended concepts for each icon, with the remaining three approaching congruency. Participants indicated that the icons were helpful when interpreting the question and that they should be more prominent.

#### Summary of User’s Responses

Although participants generally felt positively toward the summary screen, they also expressed concern that if the summary did not accord with the user’s experience, there was potential for a loss of trust in the tool, as illustrated in the following quotes:

I find it interesting just to look at the two sides, what seems to be shaping up ok, and where the struggle points are. So I think there is a bit of personal reflection that can go on the results page with these emblematic little icons, I find that quite interesting.

Male, 57

This is the make or break point, I mean if there are things here that people don’t see in their own lives, if it’s not an accurate reflection of what they answered, they’ll lose trust.

Male, 25

I was expecting more personalized results at the end, not just what I’m doing well in, something more detailed.

Female, 34

#### Risk Communication

Like their counterparts in focus group 1, many participants interpreted the information as a negative prognosis, and there was a sense that the message was impersonal and untrustworthy, as illustrated in the following quotes:

If I’m depressed, I can always find the figure down at the bottom that doesn’t get better, if I were to look at that through a dark cloud, I would see myself.

Male, 57

I mean, if somebody is depressed, you don’t want to tell them “you’re depressed, you’re a sad face.”

Male, 26

It was a bit like it wasn’t even paying attention to the answers I gave, just saying ok you’ll be ok in 3 months.

Female, 30

#### Treatment Recommendation

Participants responded positively to the treatment recommendation screens but expressed a desire for more information from the treatment recommendation regarding what they could do get better, as illustrated in the following quotes:

Yeah its very positive, isn’t it, that you can get help, that’s great.

Female, 54

So on the results page, maybe more like you should do this, take action. I mean I already know my sleep is not good but what should I do, how should I get better.

Female, 34

### Prototype 3

Given that participants in both focus groups expressed problems with the risk communication, and in the absence of a compelling alternative, we removed this element of the app entirely. Information on the treatment recommendation screen was rewritten to direct participants to specific evidence-based treatment options depending on their predicted depressive symptom severity. Treatment recommendations were based on the principles of stepped care, where the intensity of treatments increased in line with symptom severity. So, for example, patients predicted to have mild symptoms were recommended to access Internet-based self-help and psychoeducation via the myCompass program [33] and were provided with the website link.

#### First Impressions

Participants did not report any negative aspects of using the app, and all participants explicitly said it was professional and easy to use.

Consistent with focus group results, interview participants indicated that the app could raise awareness of their problems, give hope, and potentially motivate them to treatment, as illustrated in the following quotes:

It made me reflect on how my feeling have been over the past weeks and months, which did make me think, it is a bit more frequent than I thought or hoped it was.

Female, 27

**Table 3:** Summary of iterative development process (N/A: not applicable).

Themes and feedback from focus group 1		Revisions	Feedback from focus group 2	Revisions	Feedback from interviews	Revisions
**First impressions**						
	Dislike having to press “next” to navigate to next page	Remove next button	N/A	N/A	N/A	Removed a nonfunctional “tap here” button
**Iconography**						
	Mismatch in interpretation for 9/12 icons	Revise nine icons	Correct interpretation for 9/12 icons, with the remaining three approaching congruency	N/A	Icons viewed as helpful for people with English as a second language	N/A
**Summary of responses**						
	Seeing only where things are difficult could be detrimental	Include feedback on both “difficult” areas and areas that “seem to be ok for you right now”	Desire for more personalized results	None; app should be administered in a health care setting where responses can be discussed	Seeing “difficult” and “OK” areas useful for patients with depression—counteracts overgeneralization that everything is difficult	N/A
**Risk communication**						
	Focus on negative message	None; reassess in focus group 2	Focus on negative message	Removed entirely from app	N/A	N/A
**Treatment recommendation**						
	Need action-oriented message	Add recommendation to review available resources through online portal	Need more tailored recommendation	Revise recommendation to direct to specific evidence-based treatment, matched to predicted depressive symptom severity	Like being provided treatment option	Minor changes to phrasing

It made me think about how could I get more help.

Female, 36

It would be a motivation, or maybe an opportunity.

Male, 35

#### Iconography

The iconography was seen to be a seamless part of the app that could enhance understanding of the clinical prediction tool items. No participant commented on a mismatch between icon and question concept, as illustrated in the following quotes:

Yes the pictures make sense.

Female, 36

Seems pretty clear, and for people with English as a second language the infographics would help if they can’t understand the more complex words.

Male, 35

#### Summary of User’s Responses

Refinements to the feedback and treatment recommendation and removal of risk communication in response to the focus groups appeared to improve the match between the app and participant needs. Although focus group participants felt the combination of the clinical prediction tool results and risk communication left them focused on the negatives, interview participants felt positively about the feedback and recommendation, as they felt it provided solutions and a way of moving forward, as illustrated in the following quotes:

This would feel pretty good...the fact that it offers options.

Female, 34

I’d be looking forward to the [treatment recommendation], to see what options were available, of more information.

Male, 35

### The Fourth and Final App

The individual interviews identified only minimal changes in phrasing of the treatment recommendations and in one technical aspect of the app (a nonfunctional tap here button). These corrections were made in the final app. Table 3 provides a summary of participant feedback and subsequent revisions that resulted in the final app.

## Discussion

### Principal Findings

In this paper, we describe the process of user involvement in the iterative development of an app designed to estimate prognosis and guide treatment choice for patients with current depressive symptoms (using the *diamond* clinical prediction tool). We tested three prototypes with potential end users, with the feedback on each prototype used to develop the next, and ultimately, the final app. Our process of iterative development with potential end users allowed us to make improvements to the content and design of the app. We addressed initial mismatches between clinical prediction tool item content and iconography so that the icons enhanced, rather than detracted, from understanding the clinical prediction tool items. Participants indicated that the app could encourage self-reflection, provide hope, and motivate them to engage with treatment. Risk communication was identified as a significant problem and therefore removed entirely from the final app. Finally, through this process, we identified user need for personalized treatment recommendations and developed this component for the final version of the app.

### Relationship With Other Literature

This study is the first to our knowledge to use explicit user-centered design principles in development of an app-based clinical prediction tool for mental health in primary care. The long-standing problem of engaging patients in mental health treatment has not to date been solved by the advancement of health technologies such as apps, due in part to limited engagement with the technologies themselves. User-centered development has been posited to address this issue by leading to more acceptable, usable, and effective mental health technologies. For example, a lifestyle and mental health screening tool developed using a user-centered approach was deemed acceptable and usable by end users [34]. In severe mental illness, user involvement in the design of a mobile app for supporting mental health was seen to be critical in generating a product that could provide a positive user experience [35]. User-centered design has been used in development of an effective online depression prevention intervention [23] and two effective chronic pain treatment interventions [36,37].

As discussed above, Norman’s formative theory of user-centered design suggests that an individual’s interaction with a product can be conceived of as three levels of processing: visceral, behavioral, and reflective [27]. Our results show that visceral and behavioral processing did not detract from the user experience of the app, which appeared seamless. However, although ease of use has been shown to improve implementation of software [38], it is reflective processing that is most critical for adoption and use of a product. If users are to adopt and integrate technology in meaningful ways into their lives, fulfilling their emotional expectations is critical [27]. Factors related to emotions and motivations are often neglected, however, with software developers focusing predominantly on the work processes that are required and how they will take place [39]. As we increasingly seek to identify, assess, and treat mental health problems using apps and other technologies, it is critical that ease of use is not the only consideration. The results of this study suggest that involving end users in the development process can result in an app that supports meaningful reflective processing, including prompting further consideration of the symptoms and treatment in question.

### Risk Communication

Although the majority of participants in this study did not suffer from depression, they consistently interpreted the communication of their risk for persistent depression through a negative lens. As far as we are aware, this is the first study to have examined how best to communicate risk for depression. Given the negative biases inherent in many psychiatric illnesses, it is possible that the requirements for effectively communicating risk for these conditions may be very different to those for chronic physical conditions (including, eg, genetic disorders and cancer), which have to date received most attention in the risk communication field.

High-quality communication is considered an important component of shared decision making [40]. However, the challenges in communicating risk effectively are widely recognized; in their systematic review, Zipkin et al [41] acknowledge that there is likely no single best approach but put forward several recommendations on how to present risk messages. Although many of these recommendations were followed in this study (eg, using visual aids and positive framing and avoiding use of qualitative risk descriptors alone), others were not (eg, using a denominator of 1000 participants), leaving open the possibility that there may be alternative, more acceptable ways of presenting risk for persistent depression.

Given that risk communication has been shown to be highly influential on patient decision making [42], further examination of how, if at all, risk for depression and other mental health conditions can be communicated may assist in improving treatment uptake and adherence.

### Personalized Treatment Recommendation

During our development process, it became apparent that participants were more interested in the app as an action-oriented rather than informational tool. They desired a tailored treatment recommendation based on their individual symptoms, rather than information on their risk of persistent depression, even when this provided generic advice on the benefits of help-seeking (ie, “7 out of 10 people with extra help from a GP or health professional feel better”). This finding is consistent with research showing that patients with mental illness desire personalized information about available treatment [43]. Furthermore, tailored treatment recommendations have been shown to enhance patient engagement in their own care and improve adherence to treatment [44].

Importantly, participants in this trial were free to respond to the app however they chose, and the treatment recommendation we presented in prototype 3 was designed to identify the acceptability of this action-oriented message relative to the more passive information presented in prototypes 1 and 2 and not to provide specific treatment advice. Although our findings suggest this solution-focused approach was preferred, we did not set out to test how best to personalize treatment recommendations for depression. This is likely to be an important area of future investigation; although several models of personalized care have been proposed, particularly in the fields of cancer [45] and diabetes [46], there is currently a dearth of evidence suggesting how mental health treatment may best be tailored.

### Strengths and Limitations

We employed an iterative development process that allowed us to improve the app on a step-by-step basis. This allowed us to track shortcomings at each stage and avoid any flow on effects by making appropriate changes to the app as we became aware of them. We were also able to add new functions to the app as suggested by participants in the focus groups.

Our use of qualitative methodologies is also a strength of this study. Focus groups are a valuable way to generate information about what a group of people think is important and how they understand a problem [47]. Thematic analysis of interview and focus group data is an appropriate method for generating explanations of phenomena that are directly relevant for the group at study [48]. Our use of audio recordings and verbatim transcription and our use of multiple coding that engaged independent researchers in cross-checking of coding and interpretation strengthens the reliability of our results [49].

There is a risk for a biased sample in both our focus groups and our interviews. Participants responded to recruitment advertising in the community that made clear the focus on mental health and mobile apps, so it is likely we recruited primarily individuals with interest in or experience of one or both of these topics. Additionally, we recruited in an area with a highly-educated, urban population, and therefore, our recruited population may not reflect the demographics of all end users of the app.

### Future Research

The app developed in this study is being used in a randomized controlled trial to identify whether delivering the *diamond* clinical prediction tool and providing feedback and treatment recommendations in this way can improve depressive symptom severity in primary care patients, relative to usual care [50].

### Conclusions

In this study, we described how an iterative, user-centered design process led to an easy to use, engaging, and motivating app that assists in assessing prognosis and guiding treatment choice for patients with depressive symptoms. Future initiatives aimed at improving engagement with mental health assessment or treatment may consider digital apps as a platform of delivery.

## Abbreviations

eHealth: electronic health
GP: general practitioner
